# Mabs, a suite of tools for gene-informed genome assembly

**DOI:** 10.1186/s12859-023-05499-3

**Published:** 2023-10-04

**Authors:** Mikhail I. Schelkunov

**Affiliations:** Institute for Information Transmission Problems, Moscow, Russia

**Keywords:** Genome assembler, Genome assembly, Genome misassembly, Haplotypic duplications

## Abstract

**Background:**

Despite constantly improving genome sequencing methods, error-free eukaryotic genome assembly has not yet been achieved. Among other kinds of problems of eukaryotic genome assembly are so-called "haplotypic duplications", which may manifest themselves as cases of alleles being mistakenly assembled as paralogues. Haplotypic duplications are dangerous because they create illusions of gene family expansions and, thus, may lead scientists to incorrect conclusions about genome evolution and functioning.

**Results:**

Here, I present Mabs, a suite of tools that serve as parameter optimizers of the popular genome assemblers Hifiasm and Flye. By optimizing the parameters of Hifiasm and Flye, Mabs tries to create genome assemblies with the genes assembled as accurately as possible. Tests on 6 eukaryotic genomes showed that in 6 out of 6 cases, Mabs created assemblies with more accurately assembled genes than those generated by Hifiasm and Flye when they were run with default parameters. When assemblies of Mabs, Hifiasm and Flye were postprocessed by a popular tool for haplotypic duplication removal, Purge_dups, genes were better assembled by Mabs in 5 out of 6 cases.

**Conclusions:**

Mabs is useful for making high-quality genome assemblies. It is available at https://github.com/shelkmike/Mabs

**Supplementary Information:**

The online version contains supplementary material available at 10.1186/s12859-023-05499-3.

## Background

In recent years, sequencing technologies have improved significantly. Reads of Oxford Nanopore Technologies have become longer and more accurate [1], as have HiFi reads of PacBio [2, 3]. Despite this progress, genome assemblies still suffer from a number of problems, among the major of which are:

1. Fragmentation owing to long repeats with similar copies [4, 5].
2. Contamination [6, 7].
3. Haplotypic duplications [8, 9].

The latter problem is a case where, during assembly of a diploid or polyploid genome, a genome assembler mistakes corresponding regions of two homologous chromosomes with regions that originated from segmental duplications. For example, two alleles of the same gene may be mistaken for paralogues. Haplotypic duplications are dangerous because they may lead to incorrect scientific conclusions about the gene content of a genome. When two alleles are assembled separately as paralogues, an illusion of a gene duplication event is created. In highly heterozygous genomes, haplotypic duplications are so frequent that they can result in such false duplicates for thousands of genes [8, 9].

One way to address haplotypic duplications is to minimize them during the process of genome assembly. For example, authors of the genome assembler Hifiasm endowed it with a special algorithm that distinguishes corresponding regions of homologous chromosomes and segmental duplications [10]. An alternative method is to try to remove haplotypic duplications after assembly. This method is implemented in specialized programs, such as Purge_dups [8], Purge_haplotigs [11] and HapSolo [12]. However, there are no methods or combinations of methods that are 100% effective in the removal of haplotypic duplications.

To address the problem of haplotypic duplications, I created a suite of tools called "Mabs" that I describe in this article. The main two components of Mabs are Mabs-hifiasm and Mabs-flye, which serve as parameter optimizers of the popular genome assemblers Hifiasm [10] and Flye [13]. Mabs-hifiasm is intended for assembly using PacBio HiFi (also known as PacBio CCS) reads, while Mabs-flye is intended for assembly using reads of more error-prone technologies, namely, Oxford Nanopore Technologies and PacBio CLR. By optimizing the parameters of Hifiasm or Flye, Mabs reduces the number of haplotypic duplications.

## Implementation

### The metric of assembly quality used by Mabs

#### General considerations

When optimizing the parameters of a genome assembler, it is important to reasonably select a metric of assembly quality that will be maximized during optimization. Many methods for evaluating the quality of a genome assembly exist, the most popular of which are probably calculating the N50 value and performing BUSCO analyses.

#### N50

N50 is calculated as the length of the largest contig (or scaffold) such that it and all contigs (or scaffolds) longer than it constitute at least half of the sum of the lengths of all contigs (or scaffolds). Basically, N50 is a metric of contig length. The downside of N50 is that genome assemblers sometimes make improper junctions, joining sequences when they should not be joined, thus inflating N50. A parameter optimizer that maximizes N50 will favour such improper junctions.

#### BUSCO results

BUSCO is a program that is provided with many taxon-specific datasets [14]. Each of these datasets contains information about orthogroups (I refer to them as "BUSCO orthogroups") that have only one gene (I refer to them as "BUSCO genes") in genomes of at least 90% of species from a reference set of species of this taxon. An assumption on which a BUSCO analysis of the quality of a newly studied genome is based is that most BUSCO orthogroups will likely have a single gene. Thus, the number of BUSCO genes found in a genome may serve as a metric of assembly quality.

For example, the BUSCO dataset for land plants (embryophyta_odb10.2020–09-10) consists of 1614 orthogroups and was made based on a reference set of genomes of 50 species from this taxon.

BUSCO classifies orthogroups into 5 categories (see Table 1).

**Table 1:** Orthogroup categories used by BUSCO

Category	One-letter abbreviation	Meaning
Single-copy	S	Orthogroups that have a single completely assembled gene in the studied genome. A gene is considered completely assembled if its protein passed two criteria: (a) The criterion for sequence similarity to reference BUSCO proteins (b) The criterion for minimum length
Duplicated	D	Orthogroups that have more than one completely assembled gene in the studied genome. Criteria for completeness are the same as for "S"
Fragmented	F	Orthogroups that contain only genes that do not pass the criterion "(b)" but pass the criterion "(a)". Presence of such genes may be indicative of misassemblies that have led to gene fragmentation. Genes that were assembled correctly (i.e. were not fragmented) but are much shorter than reference genes also belong to this category
Missing	M	Orthogroups for which no genes passing the criterion "a)" were found
Complete	C	A compound category that is composed of orthogroups from the category "S" and orthogroups from the category "D" together

Below, I use a designation of N("S") for the number of orthogroups in category "S", and designations for the other four categories follow the same naming structure. The number of orthogroups in each of the five categories can serve as a separate metric of genome assembly accuracy. Of these five metrics, the metric most often used for genome assembly assessment is probably N("C").

However, the existence of haplotypic duplications does not decrease N("C"), since category "D" is part of category "C". When dealing with the problem of haplotypic duplications, a better method is to maximize N("S") rather than N("C"), since haplotypic duplications move orthogroups from "S" to "D", thus decreasing N("S").

The maximization of N("S") has a disadvantage because it favours assemblies where paralogues are merged. Indeed, if a genome assembler improperly merges paralogues into a single gene, this will lead to an increase in N("S"), while N("D") decreases and N("C") does not change. To address this problem, I created a novel metric that I call AG, which is an abbreviation for "the number of Accurately assembled Genes".

#### AG

Hereafter, "multicopy orthogroups" refers to what authors of BUSCO call "duplicated orthogroups". In my opinion, "multicopy orthogroups" is a better term since orthogroups in the BUSCO category "D" sometimes contain more than two genes.

**Fig. 1:**
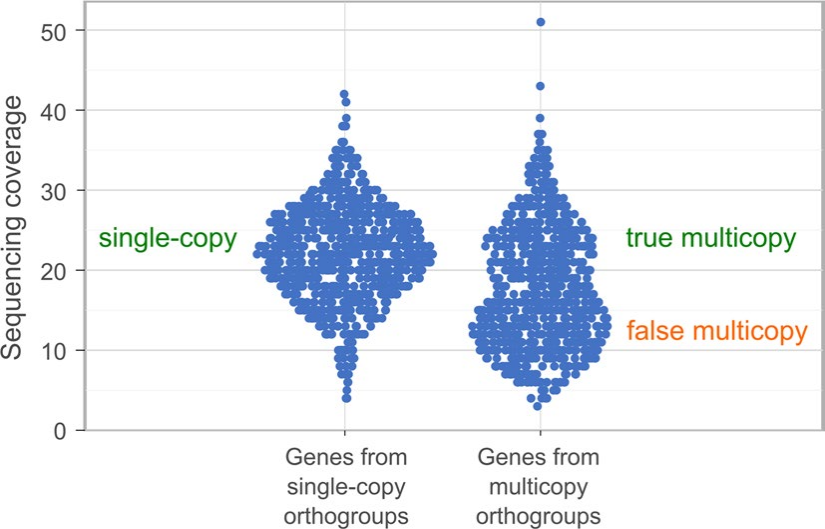
Example of a sinaplot [15] demonstrating separation of multicopy genes into true multicopy genes and false multicopy genes based on sequencing coverage

The idea behind AG is that multicopy orthogroups (orthogroups from the BUSCO category "D") may be classified into true multicopy (I designate them "TM") and false multicopy (I designate them "FM") based on their coverage. Genome assemblers are usually made in such a way that they collapse two alleles into a single sequence during the process of genome assembly. If an allele is uncollapsed (i.e., a haplotypic duplication has occurred), then the read coverage of genes of this multicopy orthogroup will be twice as low as expected. This allows differentiating true multicopy (i.e., composed of paralogues) and false multicopy (i.e., composed of uncollapsed alleles) orthogroups, see Fig. 1. AG is calculated as a sum of the following two values:

1. The number of genes in single-copy ("S") orthogroups.
2. The number of genes in true multicopy ("TM") orthogroups.

AG is not a sum of the numbers of orthogroups but a sum of the numbers of genes in them. This is because if only one gene is assembled in an orthogroup that has two paralogues, the number of orthogroups will not change (one orthogroup moves from "TM" to "S"), but the number of accurately assembled genes decreases by 1 (the number of "TM" genes decreases by 2 and the number of "S" genes increases by 1). Thus, basing AG on the number of genes in correctly assembled orthogroups is better than basing AG on the number of correctly assembled orthogroups itself.

**Fig. 2:**
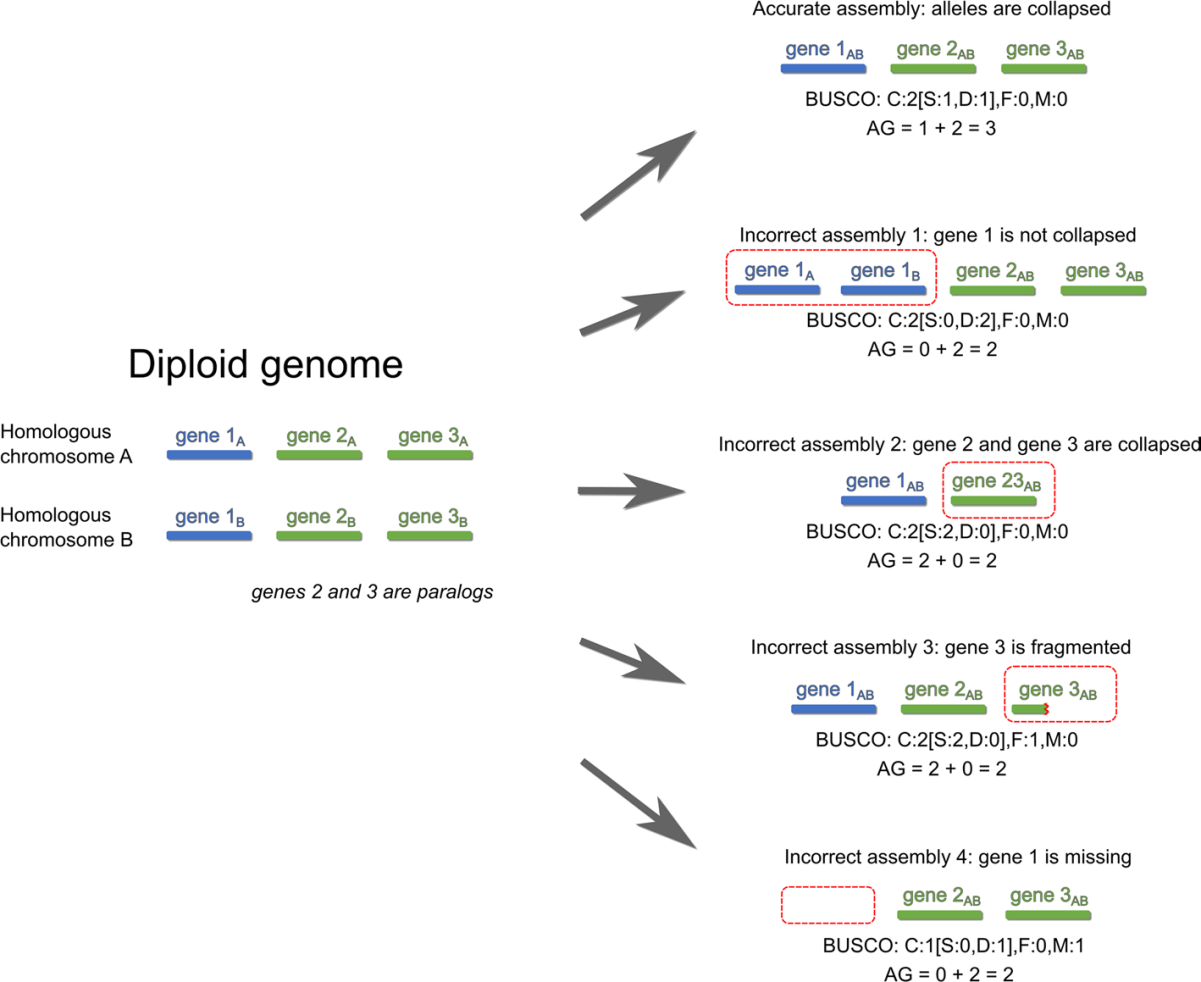
Several types of gene misassembly and their effect on AG and on BUSCO results. In this simplified diagram three BUSCO genes are depicted, two of them are paralogues. Misassemblies are marked by red dashed lines. BUSCO completeness ("C") is equal in the first four assemblies; however, only one of these assemblies is correct. At the same time, the largest AG clearly defines the best assembly. Other variants of gene misassembly are also possible, but not shown

For a comparison of AG and BUSCO statistics, see Fig. 2.

#### How AG is calculated

Given a set of reads and a genome assembly, AG is calculated follows:

1. BUSCO genes in a genome are predicted using a method that is, basically, a simplified version of the method used by BUSCO. I intentionally simplified the technique of BUSCO to increase the speed of prediction at the cost of slightly decreased accuracy. The prediction is performed as follows:
  - Potential BUSCO genes are predicted in the assembly by MetaEuk [16] using "ancestral" BUSCO proteins as a reference. The ancestral BUSCO proteins are reconstructed proteins of the last common ancestor of the species used to form the BUSCO dataset (for example, the last common ancestor of the 50 plant species of the dataset for land plants mentioned above). Sequences of the ancestral proteins are provided with each BUSCO dataset. The use of ancestral proteins as a reference to search for genes in modern species is beneficial because they are approximately equidistant to all modern species if the mutation accumulation rate did not differ greatly among lineages during evolution. In contrast, proteins of some modern genomes may be less suitable as a reference, since the phylogenetic distance to the genome under analysis may be larger and, thus, sequence similarity may be lower, making protein-to-genome alignment more difficult.
  - Proteins of potential BUSCO genes predicted by MetaEuk are compared with profile Markov models of reference proteins from BUSCO orthogroups. This comparison is performed by the program "hmmsearch" from the HMMER suite of programs [17].
  - To discriminate genes of BUSCO orthogroups from distant homologues, Mabs uses the same two criteria with the same threshold values as BUSCO: a) the criterion for sequence similarity to reference BUSCO proteins based on bit scores calculated by HMMER as described above and b) the criterion for minimum length.
2. Long reads are aligned to the genome by Minimap 2 [18].
3. Sequencing coverage in exons of all identified BUSCO genes is calculated. Mabs does not calculate coverage in introns because introns may contain transposable elements. Copies of transposable elements may be assembled incorrectly in other regions of the genome, which may lead to distorted read coverage in introns. Distorted read coverage, in turn, may decrease the accuracy of classification of multicopy orthogroups into true multicopy and false multicopy.
4. The median coverage is calculated for all single-copy orthogroups. I denote it as Cov(S).
5. For each multicopy BUSCO orthogroup, an average value between median exonic coverages of all its genes is calculated. Since true multicopy orthogroups are likely to have coverage approximately equal to Cov(S) and false multicopy orthogroups (originating from haplotypic duplications) are likely to have coverage approximately equal to Cov(S)/2, Mabs uses a threshold of (3/4) × Cov(S) to discriminate true multicopy orthogroups from false multicopy orthogroups.
  Actually, the coverage distribution of genes with average coverage Cov(S)/2 may be narrower than the coverage distribution of genes with average coverage Cov(S) if the distribution behaves similarly to the Poisson distribution, where variance increases with increasing average. Hence, the threshold should probably be somewhat lower than (3/4) × Cov(S). The threshold was set to (3/4) × Cov(S) for simplicity.
6. AG is calculated as the sum of the number of genes in single-copy orthogroups and the number of genes in true multicopy orthogroups.

Some BUSCO datasets are composed of a very large number of orthogroups. For example, the dataset for primates (primates_odb10.2021–02-19) contains 13,780 orthogroups. Searching for genes of all these orthogroups in an assembly is time-consuming. At the same time, to estimate the quality of a genome assembly, a smaller number of orthogroups is probably sufficient. Hence, to save time, for any dataset that contains more than 1000 orthogroups, Mabs by default uses only 1000 orthogroups with the most conserved sequences. Orthogroups with the most conserved sequences are determined as orthogroups with the least mean positional relative entropy, as calculated by the program "hmmstat" from the HMMER suite of programs. The use of orthogroups with conserved sequences is preferential for genome assembly quality evaluation because it decreases the chance of genes not being identified because of too diverged sequences. Mabs has an option "–number_of_busco_orthogroups" that allows a user to set the number of BUSCO orthogroups to a value other than 1000.

### Which parameters to optimize?

#### General considerations

When making a parameter optimizer for a program, it is important to choose which parameters will be optimized.

From one perspective, optimizing too many parameters at the same time requires exploration of a multidimensional space of parameters, which demands considerable time. Exploration of a multidimensional space of parameters of a genome assembler is especially time-consuming because testing a single point in the space (i.e., performing one genome assembly) may take hours or days for a eukaryotic genome; see the assembly time for Hifiasm and Flye in Tables 2 and 3 (these tables will be discussed in more detail in Results and Discussion). For especially large genomes, assembly with a relatively slow genome assembler may take months [19].

**Table 2:** Characteristics of the assemblies made by Mabs-hifiasm and Hifiasm

Species	Method of assembly	BUSCO results	N50 (bp)	Sum of contigs’ lengths (bp)	AG	Assembly time^a^	Peak RAM usage^a^
*Trifolium pratense*	Mabs-hifiasm	C:98.0%[S:92.9%,D:5.1%], F:1.3%,M:0.7%	20,490,459	460,521,547	1385	9h 50m	62 GB
	Hifiasm	C:98.1%[S:92.6%,D:5.5%], F:1.3%,M:0.6%	18,371,892	472,503,778	1380	2h 55m	54 GB
	Mabs-hifiasm + Purge_dups	C:90.7%[S:86.3%,D:4.4%], F:1.1%,M:8.2%	21,823,263	332,714,522	1279	9h 50m + 1h 41m	62 GB
	Hifiasm + Purge_dups	C:90.6%[S:86.2%,D:4.4%], F:1.4%,M:8.0%	21,823,263	317,658,493	1255	2h 55m + 1h 45m	54 GB
*Manihot esculenta*	Mabs-hifiasm	C:98.5%[S:91.0%,D:7.5%], F:0.7%,M:0.8%	34,338,427	747,732,950	1590	10h 51m	102 GB
	Hifiasm	C:98.5%[S:90.0%,D:8.5%], F:0.7%,M:0.8%	29,220,819	774,580,850	1579	4h 18m	94 GB
	Mabs-hifiasm + Purge_dups	C:46.0%[S:42.8%,D:3.2%], F:1.0%,M:53.0%	33,797,513	208,140,594	658	10h 51m + 5h 7m	102 GB
	Hifiasm + Purge_dups	C:80.5%[S:74.8%,D:5.7%], F:0.8%,M:18.7%	31,723,266	455,473,424	1250	4h 18m + 5h 51m	94 GB
*Heracleum sosnowskyi*	Mabs-hifiasm	C:98.0%[S:89.6%,D:8.4%], F:0.4%,M:1.6%	22,232,970	1,631,972,638	1392	13h 45m	57 GB
	Hifiasm	C:98.3%[S:80.9%,D:17.4%], F:0.5%,M:1.2%	13,468,048	1,814,805,347	1307	4h 8m	74 GB
	Mabs-hifiasm + Purge_dups	C:11.4%[S:10.8%,D:0.6%], F:0.9%,M:87.7%	55,489,431	178,451,278	128	13h 45m + 12h 33m	57 GB
	Hifiasm + Purge_dups	C:35.7%[S:34.0%,D:1.7%], F:0.8%,M:63.5%	12,038,317	460,607,932	453	4h 8m + 16h 32m	74 GB

^a^ Genomes were assembled using 50 threads of Intel Xeon E7-4830 CPUs

**Table 3:** Characteristics of the assemblies made by Mabs-flye and Flye

Species	Method of assembly	BUSCO results	N50 (bp)	Sum of contigs’ lengths (bp)	AG	Assembly time^a^	Peak RAM usage^a^
*Myripristis murdjan*	Mabs-flye	C:97.2%[S:95.7%,D:1.5%], F:1.1%,M:1.7%	1,738,191	849,324,108	2454	53h 14m	18 GB
	Flye	C:97.9%[S:94.6%,D:3.3%], F:1.0%,M:1.1%	1,831,223	913,966,709	2437	20h 15m	25 GB
	Mabs-flye + Purge_dups	C:97.2%[S:96.1%,D:1.1%], F:1.1%,M:1.7%	1,883,475	812,749,847	2457	53h 14m + 24m	18 GB
	Flye + Purge_dups	C:97.8%[S:96.7%,D:1.1%], F:1.0%,M:1.2%	2,177,694	834,985,181	2483	20h 15m + 20m	25 GB
*Adineta vaga*	Mabs-flye	C:66.3%[S:57.0%,D:9.3%], F:10.4%,M:23.3%	2,333,984	112,876,648	163	9h 50m	32 GB
	Flye	C:67.0%[S:57.5%,D:9.5%], F:9.9%,M:23.1%	1,636,027	116,202,497	161	2h 43m	36 GB
	Mabs-flye + Purge_dups	C:66.3%[S:57.3%,D:9.0%], F:10.4%,M:23.3%	2,698,890	106,057,374	164	9h 50m + 6m	32 GB
	Flye + Purge_dups	C:66.6%[S:57.3%,D:9.3%], F:9.9%,M:23.5%	2,339,515	105,961,627	162	2h 43m + 5m	36 GB
*Mytilus coruscus*	Mabs-flye	C:85.0%[S:78.4%,D:6.6%], F:4.2%,M:10.8%	308,470	2,165,590,554	2040	114h 46m	210 GB
	Flye	C:83.7%[S:66.6%,D:17.1%], F:4.4%,M:11.9%	280,259	2,333,181,150	1726	70h 10m	216 GB
	Mabs-flye + Purge_dups	C:84.3%[S:82.0%,D:2.3%], F:4.2%,M:11.5%	343,029	1,951,757,051	2144	114h 46m + 1h 55m	210 GB
	Flye + Purge_dups	C:82.4%[S:79.8%,D:2.6%], F:4.5%,M:13.1%	320,773	1,962,315,961	2107	70h 10m + 1h 49m	216 GB

^a^Genomes were assembled using 50 threads of Intel Xeon E7-4830 CPUs

From another perspective, the more parameters are optimized, the greater the possible improvement in genome assembly.

Genome assemblers sometimes have dozens of parameters that affect their algorithm. The most prominent example that I have seen is Shasta [20], with Shasta 0.10.0 having 116 parameters that may affect the produced assembly.

#### Hifiasm

For Hifiasm, the choice of a parameter for optimization is straightforward. It is the parameter "-s" that regulates the work of a special algorithm of Hifiasm made specifically to address haplotypic duplications. "-s" can have values in the range of 0 to 1. The default "-s" value in Hifiasm is 0.55, except when performing trio binning (usage of reads of both parents of the studied organism during assembly), where haplotypic duplication removal is not used. The algorithm behind "-s" is described in the work of Cheng et al. [10], but speaking simply, the closer the value of "-s" is to 0, the more aggressive Hifiasm is in the removal of similar sequences from the assembly.

The sole parameter of Hifiasm that Mabs-hifiasm optimizes is "-s".

#### Flye

Optimization of Flye to reduce the number of haplotypic duplications is not as straightforward as the optimization of Hifiasm, since Flye has no parameters dedicated specifically for removal of haplotypic duplications, except for the parameter "–no-alt-contigs", which is boolean ("true" or "false"). My tests (data not provided) indicate that "–no-alt-contigs" is probably always beneficial for removal of haplotypic duplications, thus Mabs-flye always runs Flye with this option. Based on my understanding of the algorithm of Flye, I chose two parameters for optimization:

1. "assemble_ovlp_divergence". When used in combination with "assemble_divergence_relative = 0", as in Mabs-flye, the parameter "assemble_ovlp_divergence" regulates how dissimilar sequencing reads are allowed to be during disjointig construction. For a description of the algorithm and the term "disjointig" see the work of Kolmogorov et al. [13], but basically, higher values of "assemble_ovlp_divergence" may lead to more aggressive removal of similar sequences from the assembly.
2. "repeat_graph_ovlp_divergence". This parameter regulates how dissimilar sequences from disjointigs are allowed to be during merging of disjointigs into a repeat graph. As with "assemble_ovlp_divergence", larger values of this parameter may lead to more aggressive removal of similar sequences from the assembly.

Default values of these parameters in Flye differ depending on the sequencing technology used to produce the reads being assembled.

To accelerate the assembly, Mabs-flye assumes these two parameters to be equal, referring to them as a single parameter "max_divergence". Thus, the parameter optimization is performed by Mabs-flye in a unidimensional space, just as in the case with Mabs-hifiasm.

### The workflow of Mabs-hifiasm and Mabs-flye

#### General considerations

The workflow of Mabs-hifiasm is similar to the workflow of Mabs-flye. In Sect. "Hifiasm" I will describe the workflow of Mabs-hifiasm, and then, in Sect. "Flye", I will pinpoint the differences between Mabs-flye and Mabs-hifiasm.

#### Mabs-hifiasm

**Fig. 3:**
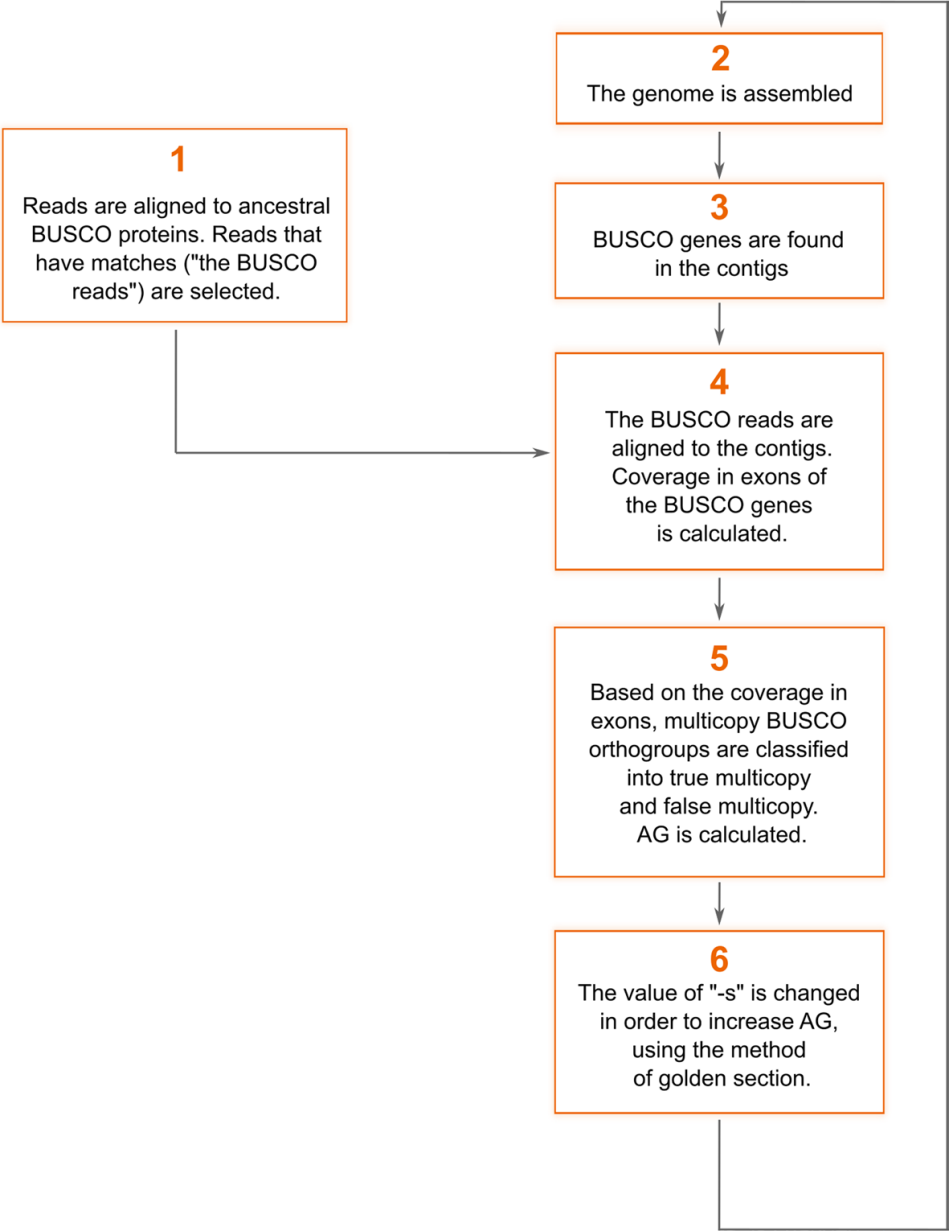
The flowchart of Mabs-hifiasm

The basic scheme of Mabs-hifiasm is provided in Fig. 3. Speaking simply, Mabs-hifiasm tries to find the value of the "-s" parameter of Hifiasm that provides as large an AG value as possible. The maximization is performed using the method of the golden section [21]. The parameter "-s" can range from 0 to 1. In the golden section method, the first two values to be examined are middle points, $(\frac{\sqrt{5}-1}{\sqrt{5}+1})$ and $(1- \frac{\sqrt{5}-1}{\sqrt{5}+1})$, while the next values are determined based on the AG values.

The basic steps in the workflow of Mabs-hifiasm are as follows:

1. Reads are aligned to ancestral BUSCO proteins by DIAMOND [22]. The purpose is to select reads that belong to BUSCO genes. Hereafter I refer to them as "BUSCO reads". They will be used in step 4 to calculate the coverage of exons of BUSCO genes. Of course, it is possible to use all reads for the calculation of coverage, but using only BUSCO reads saves time since they constitute only a portion of all reads. For large eukaryotic genomes, where most regions are intergenic or intronic, the time saved by aligning only BUSCO reads becomes especially prominent.
  Since errors in long reads are often indels [23–26] and, thus, may lead to frameshifts, DIAMOND is run with the option "--frameshift", which allows for frameshifts in the alignment.
2. A genome is assembled by Hifiasm using the current value of "-s".
3. AG is calculated as described in the Sect. "How AG is calculated". This step is represented by three boxes (from "BUSCO genes..." to "Based on the coverage of exons...") in Fig. 3.
4. Based on the AG value, the next value of "-s" is selected using the golden section method.

Ten points (including the two starting middle points) are examined by Mabs-hifiasm during the golden section optimization. Including more points may provide more accuracy at the cost of increasing assembly time. My tests show that 10 points is sufficient for determining the value of "-s" that provides the maximum or nearly maximum AG value (Additional file 1: Figs. S1 and S2).

#### Mabs-flye

Mabs-flye uses basically the same workflow as Mabs-hifiasm, with the following differences:

1. Instead of Hifiasm, Mabs-flye uses Flye as the genome assembler. The need for two separate tools (Mabs-hifiasm and Mabs-flye) appeared because the algorithm of Hifiasm is intended foremost for very accurate (PacBio HiFi) reads, while the algorithm of Flye is intended mainly for considerably less accurate (PacBio CLR or Oxford Nanopore) reads. As of 2023, both PacBio HiFi and Oxford Nanopore technologies are widely used; thus, Mabs is split into Mabs-hifiasm and Mabs-flye. Although Flye has a dedicated option that allows it to assemble PacBio HiFi reads, "–pacbio-hifi", my tests on several genomes (data not provided) suggest that Hifiasm usually assembles genomes from HiFi reads better than Flye does.
  Taking into account the currently increasing accuracy of Oxford Nanopore reads [1, 27], it is possible that in the future, Hifiasm will also be suitable for Oxford Nanopore reads, thus reducing the necessity for Flye and, consequently, for Mabs-flye.
2. While Mabs-hifiasm optimizes the parameter "-s" of Hifiasm, Mabs-flye optimizes the parameter that I call "max_divergence"; see the Sect. "Which parameters to optimize?".
3. While the optimization of "-s" by Mabs-hifiasm is performed directly, Mabs-flye log-transforms "max_divergence" and performs the golden section optimization for log_10_("max_divergence") due to the nature of "max_divergence". Basically, if a user provides Mabs-flye with very accurate reads (for example, with an error rate of approximately 1%), then fine-tuning of "max_divergence" may be beneficial. On the other hand, if a user provides Mabs-flye with highly inaccurate reads (for example, with an error rate of approximately 15%), then the parameter tuning should be more "coarse-grained". This is achieved by logarithmically transforming "max_divergence". While the interval of "-s" is [0; 1], the interval of "max_divergence" examined by Mabs is [0.0001; 0.5] or, in other words, [0.01%; 50%].
4. When optimizing "-s", Mabs-hifiasm assembles the whole genome, but Mabs-flye assembles only genes.
  Hifiasm is a fast assembler, especially taking into account that it can reuse intermediate files to produce an assembly with another "-s". On the other hand, Flye needs to perform an assembly for each "max_divergence" from the very beginning, which makes it slow. To address this problem of Flye, Mabs-flye uses only "BUSCO reads" (for the definition, see above) during assembly. Thus, Mabs-flye assembles only genes and evaluates AG only for genes. When the optimal "max_divergence" is found, Mabs-flye performs the final assembly, this time using all reads.
  Assembling only genes may have a potential downside since BUSCO reads are reads that align to ancestral BUSCO proteins; thus, if the genome being assembled has very long introns and reads used for assembly are relatively short, introns may not be fully covered. This will lead to fragmentation of genes in the assembly of Mabs-flye, which in turn leads to problems in properly calculating the number of BUSCO genes and, thus, detrimentally affects the calculation of AG. However, typical genomic Oxford Nanopore reads in 2023 have lengths about 10–100 kbp, which is probably more than the typical length of eukaryotic introns [28]. Thus, assembling only genes to find the optimal "max_divergence" is probably rational.
5. In contrast to Mabs-hifiasm, Mabs-flye polishes genes using Proovframe.
  A problem with error-prone reads is that an assembly made from them will also have many errors. Such errors are usually insertions or deletions of several bases [29–31]. As they occur in CDSs, they likely lead to frameshifts, thus harming the ability of Mabs to find BUSCO genes and, thus, to calculate AG.
  One way to address this problem is to polish the assembly with accurate short reads. Alignment-based polishers, such as Racon [32], Pilon [33] and POLCA [34], will considerably increase the computational time of Mabs-flye since polishing is required for each of the 10 points tested by the golden section method, and read alignment is a time-consuming operation. An alternative is to use alignment-free polishers, such as ntEdit [35], which are faster. However, alignment-free polishing is not as accurate as alignment-based polishing.
  A simple alternative is to use pseudopolishing by Proovframe [36]. Proovframe is a tool that aligns reference proteins to a genome assembly and fixes in-frame stop-codons and frameshifts that break open reading frames. The utilization of Proovframe allows to fix frameshifting assembly errors, thus making the detection of BUSCO genes possible. Mabs uses ancestral BUSCO proteins as the reference for Proovframe.
  Polishing by Proovframe has a downside in that the actual genome sequence may be distorted since it is fixed based on sequences of ancestral proteins that likely differ from sequences of current proteins of this species. For example, a frameshift in an actual pseudogene in a studied genome will be removed by Proovframe. For this reason, when the optimal "max_divergence" has been determined and Mabs-flye makes the *final* assembly using *all* reads, Proovframe is not used.

#### Approaches used to accelerate Mabs

A simple implementation of Mabs may have looked as follows:

1. Run a genome assembler many times with different values of its parameters.
2. If error-prone (Oxford Nanopore or PacBio CLR) reads were used, polish each assembly using short accurate reads.
3. Map all reads to each assembly and calculate the coverage of BUSCO genes.
4. Based on the coverage, calculate AG and determine the best assembly.

This approach may be inefficient because assembly and polishing of a large genome may take days. Here I outline the main techniques that Mabs uses to accelerate the process:

1. Re-use of intermediate Hifiasm files.
  Hifiasm is able to use the same intermediate files to generate assemblies with different values of "-s". Mabs-hifiasm re-uses some files generated during the first Hifiasm assembly in all subsequent Hifiasm assemblies, thus accelerating the subsequent assemblies.
2. When Mabs-flye optimizes "max_divergence", it assembles only genes.
  When Mabs-flye starts, it finds sequencing reads that correspond to BUSCO genes ("BUSCO reads"). When Mabs-flye optimizes "max_divergence", it performs assemblies using only BUSCO reads. This accelerates the assembly because large genomes mainly consist of non-coding regions. In contrast, Mabs-hifiasm assembles the genome using all reads, because the technique "1." makes individual assemblies (except for the first one) fast.
3. The coverage of BUSCO genes is calculated using only BUSCO reads.
  When calculating the coverage of BUSCO genes, both Mabs-hifiasm and Mabs-flye map only BUSCO reads instead of mapping all reads.
4. Mabs-flye uses pseudopolishing. As noted above, the traditional polishing by short accurate reads can be slow. To make detection of BUSCO genes possible despite assembly errors, Mabs-flye uses pseudopolishing by Proovframe. Mabs-hifiasm does not require this technique because assemblies made from PacBio HiFi reads are significantly more accurate than assemblies made from Oxford Nanopore of PacBio CLR reads and do not require polishing.

### Avoiding biases during the development and testing of Mabs

#### General considerations

A number of biases may lead to exaggeration of the quality of a bioinformatic program. Here, I describe how I addressed two relatively nonobvious biases.

#### Avoiding overfitting of Mabs to specific genomes

If, during its development, a genome assembler is tested on some genomes, the algorithm of this assembler may become overfitted to produce good results for these particular genomes and these particular reads. If the assembler is then compared with other assemblers on the same genomes and reads, it may outperform them, but this will not mean that the studied assembler will outperform them for other genomes and reads.

To address this problem, during the development of Mabs-hifiasm and Mabs-flye, I tested their ability to assemble genomes other than those used for comparison with Hifiasm and Flye in this article. Namely, during the development, I used genomes of *Arabidopsis thaliana* (PacBio HiFi and Oxford Nanopore reads), *Caenorhabditis elegans* (PacBio CLR reads) and the *Fagopyrum esculentum* cultivar Dasha (PacBio HiFi and Oxford Nanopore reads).

The first two genomes are small (approximately 100 Mbp) and allow for quick testing of Mabs, although their assemblies usually had no haplotypic duplications at all. On the other hand, the genome of *Fagopyrum esculentum* represents an ideal case to test a genome assembler. During the last million years, *Fagopyrum esculentum* experienced a fast expansion of 10 kbp-long transposable elements that tripled its genome, increasing the genome size from approximately 500 Mbp to approximately 1.5 Gbp [37]. Considering the recentness of this transposable element explosion, their copies are similar to each other, thus increasing the difficulty of genome assembly. Additionally, samples of the cultivar Dasha that were used for production of PacBio HiFi and Oxford Nanopore reads had relatively high heterozygosity (approximately 4%, to be published), which also increases the complexity of the assembly. Thus, the *Fagopyrum esculentum* cultivar Dasha represents a difficult case for genome assembly and, consequently, is highly suitable for tuning genome assemblers. The creation of a high-quality assembly of *Fagopyrum esculentum* is underway.

#### Avoiding circular reasoning

**Fig. 4:**
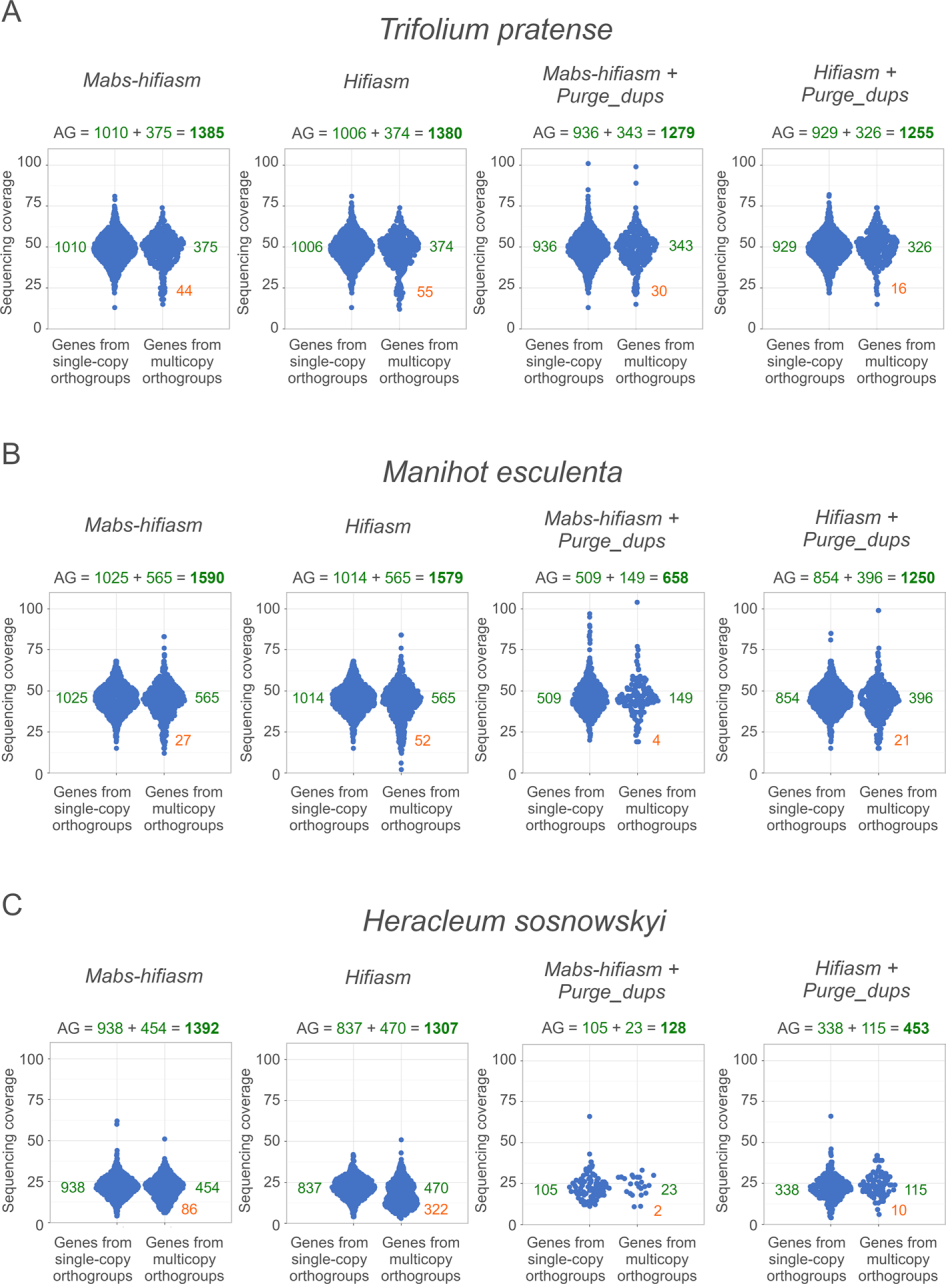
Sinaplots of gene coverage in assemblies made by Mabs-hifiasm and Hifiasm.** A** for *Trifolium pratense*, **B** for *Manihot esculenta*, **C** for *Heracleum sosnowskyi*. Each dot is a BUSCO gene

**Fig. 5:**
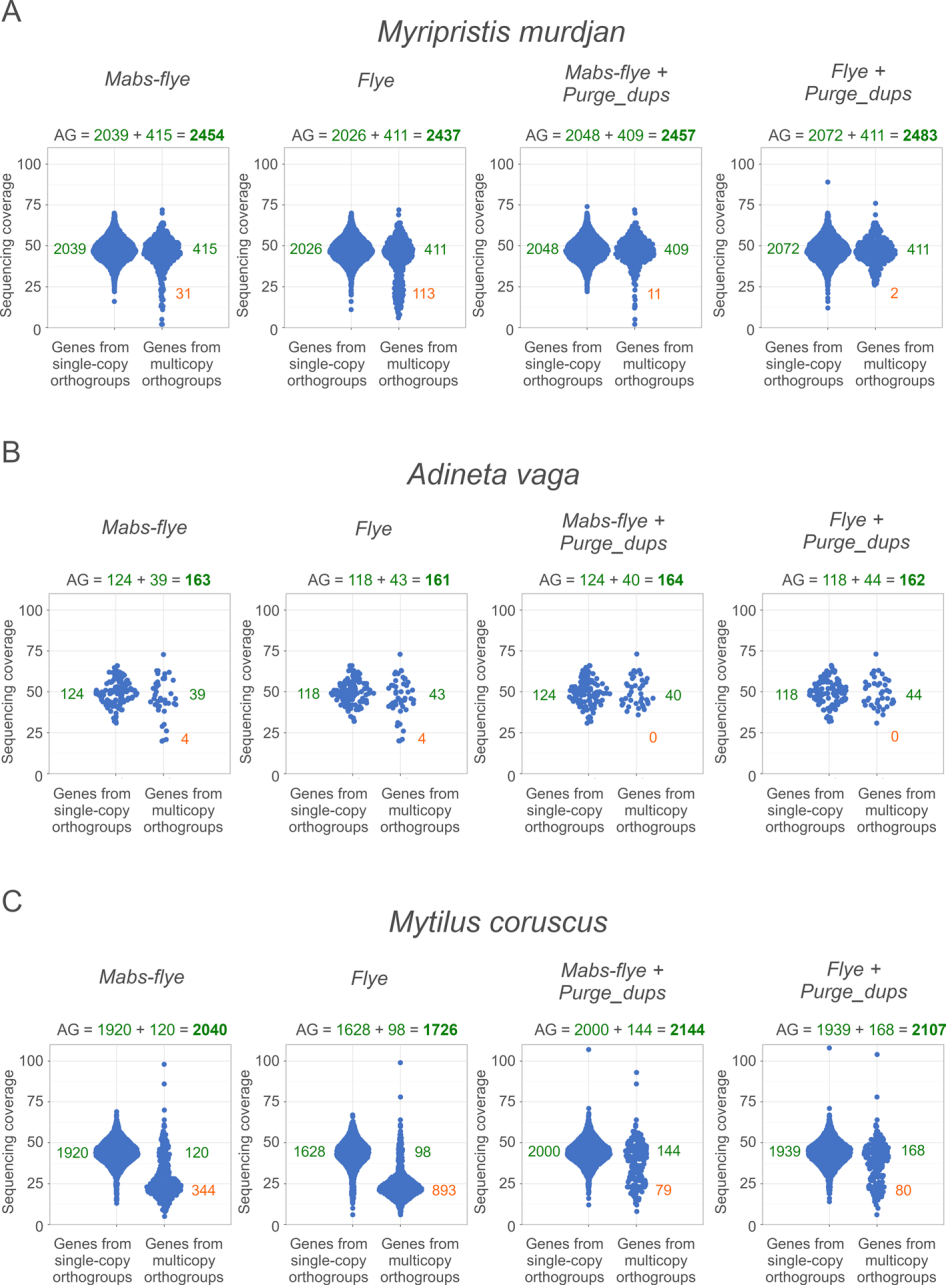
Sinaplots of gene coverage in assemblies made by Mabs-flye and Flye. Each dot is a BUSCO gene

Mabs uses the detection of BUSCO genes during assembly. The quality of assembly of BUSCO genes (i.e., "AG") is maximized by both Mabs-hifiasm and Mabs-flye. On the other hand, in Figs. 4 and 5 and in Tables 2 and 3, AG is reported as the metric of assembly quality. This may create a bias in that the same metric is used to assess the assembly as is maximized during the assembly. To address this, I split all BUSCO datasets into two parts: the first part was used by Mabs-hifiasm or Mabs-flye to calculate AG during the assembly, while the second part was used to calculate AG for Figs. 4 and 5 and perform BUSCO analyses for Tables 2 and 3 (see Table 4).

**Table 4:** Subdivision of BUSCO datasets into parts used for assembly and testing

Species	BUSCO dataset	Total number of orthogroups in the BUSCO dataset	Number of orthogroups used during the assembly	Number of orthogroups used to test the final assembly quality
*Trifolium pratense*	eudicots_odb10	2326	1000	1326
*Manihot esculenta*	eudicots_odb10	2326	1000	1326
*Heracleum sosnowskyi*	eudicots_odb10	2326	1000	1326
*Myripristis murdjan*	actinopterygii_odb10	3640	1000	2640
*Adineta vaga*	metazoa_odb10	954	500	454
*Mytilus coruscus*	mollusca_odb10	5295	1000	4295

### Genomes used for comparison of Mabs with Hifiasm and Flye

To test Mabs, I performed a literature analysis and compiled a set of 5 species for which authors reported a high number of haplotypic duplications: *Trifolium pratense* [38], *Manihot esculenta* [39], *Myripristis murdjan* [8, 40], *Adineta vaga* [41], and *Mytilus coruscus* [42, 43]. In addition, I included *Heracleum sosnowskyi*, which I studied personally and which also had many haplotypic duplications [44]. For detailed information about the genomes and their sequencing reads, see Table 5. The genomes of *Trifolium pratense*, *Manihot esculenta* and *Heracleum sosnowskyi* were sequenced using PacBio HiFi technology and were thus used to compare Mabs-hifiasm and Hifiasm. The other three genomes were sequenced using error-prone technologies and were thus used to compare Mabs-flye and Flye. All six genomes are diploid.

**Table 5:** Information on the genomes and sequencing reads used in this article

Species	Genome size, estimated without sequencing (Mbp)	Genome size, estimated as the size of the assembly with the largest N50 among published assemblies (Mbp)	Sequencing technology	Sequence Read Archive identifiers of reads	N50 of reads (bp)	Approximate genome coverage by reads^a^
*Trifolium pratense*	636 [45], 474 [46], 418 [47], 557 [48]	423 [49]	PacBio HiFi	SRR15433789	20,082	50
*Manihot esculenta*	817 [50]	706 [39]	PacBio HiFi	ERR5485301	20,363	42
*Heracleum sosnowskyi*	1751 [48]	1629 [44]	PacBio HiFi	SRR23251371, SRR23251372	14,679	22
			Illumina Hi-C, paired-end	SRR23251383, SRR23251384	76	34
*Myripristis murdjan*	no estimates	835 [51]	PacBio CLR	ERR3449630, ERR3449634, ERR3449635, ERR3453872, ERR3453873, ERR3453874, ERR3453875, ERR3453876	24,966	50
			Illumina shotgun, paired-end ^b^	ERR3655549	151	155
*Adineta vaga*	362^c^ [52]	101^c^ [53]	Oxford Nanopore	SRR13348928	38,562	50
			Illumina shotgun, paired-end ^b^	SRR13348929	251	331
*Mytilus coruscus*	1858 [54]	1567 [55]	Oxford Nanopore	ERR3415816	24,641	50
			Illumina shotgun, paired-end ^b^	ERR3431204	150	53

^a^The approximate genome coverage was calculated based on the following genome sizes: *Trifolium pratense* 450 Mbp, *Manihot esculenta* 750 Mbp, *Heracleum sosnowskyi* 1700 Mbp, *Myripristis murdjan* 850 Mbp, *Adineta vaga* 100 Mbp, *Mytilis coruscus* 1600 Mbp

^b^Used only for polishing, after the assembly

^c^The cause of the discrepancy between genome size estimates for *Adineta vaga* is unknown

### Used programs and their parameters

#### Preprocessing of reads

To accelerate the assembly, long reads that provided genome coverage above 50 were downsampled to coverage 50 by Filtlong 0.2.1 [56]. It has been demonstrated previously that increasing read coverage above 50 does not improve the assembly quality of diploid genomes substantially [41, 57–64]. Quality trimming and adapter trimming of Illumina reads were performed by Fastp 0.21.0 [65] with the following criteria:

1. Adapters were trimmed using the default method of Fastp, which does not require knowledge of adapter sequences.
2. Bases with Phred quality scores below 3 were removed from the 3'-ends.
3. If a 5 bp window in a read had an average Phred score below 15, this window and everything towards the 3'-end of the read were removed.
4. If the average Phred quality score of a read remained below 20 after the abovementioned procedures, the read and its pair were removed.
5. If after the abovementioned procedures the length of a read became less than 30 bp, the read and its pair were removed.

#### Genome assembly

Genomes were assembled with Mabs 2.11, Hifiasm 0.16.1, and Flye 2.9.1.

Hifiasm was run with default parameters. All assemblies performed by Hifiasm were made with PacBio HiFi reads, except the assembly of the genome of *Heracleum sosnowskyi*, where Hi-C reads were also used.

During assembly with Flye, PacBio CLR reads of *Myripristis murdjan* were provided with the option "–pacbio-raw", while Oxford Nanopore reads of *Adineta vaga* and *Mytilus coruscus* were provided with the option "nano-raw". The option "–no-alt-contigs", which is a special option of Flye for removal of haplotypic duplication, was always used. Other parameters of Flye were default.

For Mabs-hifiasm and Mabs-flye, paths to BUSCO datasets specified in Table 4 were provided via the option "–local_busco_dataset". By default, Mabs uses 1000 orthogroups during the assembly. The database metazoa_odb10, used for *Adineta vaga*, contains only 954 orthogroups, and a portion of them had to be used for assessing the assembly quality (see "Avoiding circular reasoning"). Hence, for *Adineta vaga,* the value of the parameter "–number_of_busco_orthogroups" was set to 500 instead of the default 1000. Similar to the Hifiasm assembly of *Heracleum sosnowskyi*, in the Mabs-hifiasm assembly of *Heracleum sosnowskyi,* Hi-C reads were used along with HiFi reads.

#### Postprocessing of assemblies

For Flye assemblies, polishing was performed by HyPo 1.0.3 [29], providing coverage values of Illumina reads (the option "–coverage-short") as indicated in Table 5. Assemblies made by Mabs-flye were polished in the same way. Hifiasm assemblies do not require polishing [10], and consequently, assemblies of Mabs-hifiasm do not require polishing either.

Deduplication was performed by Purge_dups with default parameters.

#### Quality control of assemblies

BUSCO analysis of the assemblies was performed using BUSCO 5.3.2. Datasets for the BUSCO analysis were manually constructed by excluding the orthogroups that were used by Mabs during assembly (see "Avoiding circular reasoning") from the datasets described in Table 4.

The AG values of the assemblies were calculated by calculate_AG from Mabs 2.11 using the same datasets as those used by BUSCO.

## Results and discussion

### Brief description of the algorithm of Mabs

The workflow of Mabs can be briefly described as follows:

1. Mabs makes a series of assemblies by Hifiasm or Flye, using different values of their parameters. Since the genome assembly process is time-consuming, Mabs implements several tricks to accelerate it.
2. The quality of each assembly is evaluated using a special metric that I call AG. AG is an abbreviation for "the number of Accurately assembled Genes". It is the number of assembled genes from single-copy BUSCO orthogroups plus the number of genes from true multicopy BUSCO orthogroups.
3. A distinctive feature of Mabs compared to BUSCO is that Mabs classifies multicopy (i.e. containing several genes) BUSCO orthogroups into true multicopy and false multicopy. True multicopy orthogroups consist of paralogues, while false multicopy orthogroups consist of haplotypic duplications. Mabs is able to distinguish true multicopy orthogroups from false multicopy orthogroups, because genes originating from haplotypic duplications have two times lower coverage than correctly assembled genes (Fig. 1). The assembly with the largest AG is considered the best and reported to the user.

For a more detailed description of the algorithm of Mabs, see the section Implementation.

AG is, in my opinion, a very informative metric of the genome assembly quality. In addition to Mabs-hifiasm and Mabs-flye, the Mabs suite of tools includes the third tool called calculate_AG that allows a user to calculate AG for any genome assembly. This tool can be used to compare genome assemblies created by different genome assemblers to determine which of them has the most accurately assembled genes. In contrast to BUSCO, calculate_AG is able to determine which multicopy orthogroups are true, and which are assembly errors.

One disadvantage of AG is that it is poorly suited to compare two *nearly perfect* genome assemblies. For example, the recent telomere-to-telomere human genome assembly was made for the genome of a hydatidiform mole, which has an advantage for performing genome assembly in that it is nearly 100% homozygous [5]. With HiFi reads or ultralong Oxford Nanopore Technology reads, it is possible to obtain genome assemblies that are accurate to the level of all protein-coding genes being assembled perfectly. The main problem with such assemblies is the difficulty in assembling tandem repeats with long monomers, such as centromeres and rDNA clusters [5, 63, 66]. Thus, any assembly quality metrics that are based on how well protein-coding genes are assembled, be it AG or results of BUSCO, will usually be useless for comparing two nearly perfect assemblies.

The strategy of "gene-informed parameter optimization" utilized by Mabs may be applied to any genome assembler that has some parameters that affect its algorithm. Hifiasm and Flye were chosen because in multiple articles they were shown to be the best or among the best assemblers for accurate (PacBio HiFi) and error-prone (Oxford Nanopore Technologies and PacBio CLR) reads, respectively [4, 41, 63, 67–73]. Since Hifiasm and Flye were favourably compared with other genome assemblers many times, in this work I compare Mabs only with Hifiasm and Flye and do not make comparisons with other assemblers.

### Comparison of Mabs-hifiasm with Hifiasm

Mabs-hifiasm was compared with Hifiasm on three genomes that belonged to plants *Trifolium pratense* (the red clover), *Manihot esculenta* (cassava) *Heracleum sosnowskyi* (Sosnowsky's hogweed). Genomes of these three plants were selected for the analysis because their assemblies were reported to suffer from a large number of haplotypic duplications [38, 39, 44].

As can be seen in Fig. 4 and Table 2, Mabs-hifiasm assembled genes of *Trifolium pratense* better than Hifiasm. For the 1326 BUSCO orthogroups used for the analysis (see Table 4 and the paragraph "Avoiding circular reasoning" in the section Implementation), Mabs-hifiasm assembled 1010 genes in single-copy orthogroups and 375 genes in true multicopy orthogroups, while Hifiasm assembled 1006 genes in single-copy orthogroups and 374 genes in true multicopy orthogroups. At the same time, Mabs-hifiasm assembled 44 genes in false multicopy orthogroups, while Hifiasm assembled 55 genes in false multicopy orthogroups. In other words, the number of correctly assembled genes was larger in the assembly of Mabs-hifiasm, while the number of incorrectly assembled genes was larger in the assembly of Hifiasm. BUSCO's completeness ("C") was smaller in the assembly of Mabs-hifiasm compared to the assembly of Hifiasm. However, the number of haplotypic duplications in the assembly of Mabs-hifiasm was also smaller than in the assembly of Hifiasm, which resulted in the total number of accurately assembled genes ("AG") being larger in the assembly of Mabs-hifiasm. Thus, overall, the assembly of Mabs-hifiasm is more correct. N50 was also larger in the assembly made by Mabs-hifiasm (Table 2). The use of Purge_dups had, for some unknown reason, a detrimental effect on the assemblies of both Mabs-hifiasm and Hifiasm. Though the assembly of Mabs-hifiasm was better, it required more time, taking approximately 10 h instead of 3 h for Hifiasm.

The assembly made by Mabs-hifiasm for the genome of *Manihot esculenta* also had better assembled genes than the assembly made by Hifiasm. As in the case of *Trifolium pratense*, it also had a larger N50, but required more time. Purge_dups decreased the assembly quality.

The *Heracleum sosnowskyi* genome assembly made by Mabs-hifiasm was also better than the assembly made by Hifiasm, both in terms of the gene assembly accuracy and in terms of N50. As can be observed in Fig. 4, Mabs-hifiasm incorrectly merged two pairs of paralogues. The created chimeric genes can be seen as two points on the left part of the diagram that have coverage approximately two times higher than normal single-copy genes. However, the number of correctly assembled genes in single-copy orthogroups in the assembly of Mabs-hifiasm was higher than in the assembly of Hifiasm. Also, the number of genes in false multicopy orthogroups in the assembly of Mabs-hifiasm (86) was much smaller than in the assembly of Hifiasm (322). The number of genes in false multicopy orthogroups in the assembly of Hifiasm was so large that the coverage distribution of multicopy orthogroups is noticeably bimodal. As with the genomes of *Trifolium pratense* and *Manihot esculenta*, the usage of Purge_dups had a detrimental effect on the assemblies made by Hifiasm and Mabs-hifiasm.

Overall, for all three genomes Mabs-hifiasm made better assemblies than Hifiasm. The assemblies of Mabs-hifiasm were better both in terms of gene assembly accuracy and in terms of N50. However, on average, Mabs-hifiasm was approximately three times slower than Hifiasm.

### Comparison of Mabs-flye with Flye

Mabs-flye was compared with Flye on the genomes of *Myripristis murdjan* (a species of soldierfish), *Adineta vaga* (a species of rotifers) and *Mytilus coruscus* (the Korean mussel). These three genomes were selected because their assemblies were previously reported to suffer from a lot of haplotypic duplications [8, 40–43].

For *Myripristis murdjan*, Mabs-flye assembled genes more accurately than Flye (Fig. 5, Table 3). The number of genes in single-copy orthogroups in the assembly made by Mabs-flye was 2039, while in the assembly made by Flye it was 2026. The number of genes in true multicopy orthogroups in the assembly made by Mabs-flye was 415, while in the assembly made by Flye it was 411. The number of genes in false multicopy orthogroups in the assembly made by Mabs-flye was 31, while in the assembly made by Flye it was 113. Purge_dups improved both assemblies, which can be seen from the increase in AG. For the assembly of Flye this increase was larger, which lead to the assembly of Flye + Purge_dups being better than the assembly of Mabs-flye + Purge_dups. N50 was better in the assembly made by Flye than in the assembly made by Mabs-flye. Mabs took approximately 2.5 times longer than Mabs-flye. Overall, if taking into account the assembly improvement by Purge_dups, the genome of *Myripristis murdjan* was better assembled by Flye than by Mabs-flye.

For *Adineta vaga*, the assembly of Mabs-flye was better than the assembly made by Flye both before and after the usage of Purge_dups, both in terms of gene assembly accuracy and in terms of N50. The assembly by Mabs-flye took approximately 3.5 more time than the assembly by Flye. The low number of BUSCO genes found in the genome of *Adineta vaga* is partially explained by the low (454) number of orthogroups used for the analysis (Table 4) and partially by the high phylogenetic distance between the last common ancestor of animals and *Adineta vaga*, which results in low sensitivity of the detection of BUSCO genes because of the low sequence similarity.

For *Mytilus coruscus* Mabs-flye made a better assembly than Flye. However, the number of haplotypic duplications in the assembly made by Mabs-flye was large, though smaller than in the assembly made by Flye (Fig. 5). The application of Purge_dups significantly improved both assemblies, but the assembly made by Mabs-flye was still better.

Overall, for two of the three genomes (those of *Adineta vaga* and *Mytilus coruscus*) Mabs-flye made better assemblies than Flye. For the genome of *Myripristis murdjan* the assembly made by Flye became better than the assembly made by Mabs-flye after the use of Purge_dups. While Purge_dups had a detrimental effect on all assemblies made by Mabs-hifiasm and Hifiasm, its effect on the assemblies made by Mabs-flye and Flye was always positive. The cause of this is unclear.

## Conclusions

In this article I described the suite of tools Mabs, which consists of Mabs-hifiasm and Mabs-flye. Mabs-hifiasm and Mabs-flye optimize parameters of genome assemblers Hifiasm and Flye, trying to make assemblies where genes are assembled better than when Hifiasm and Flye are run with default parameters. For five of the six tested genomes Mabs created better assemblies than Hifiasm and Flye at the cost of approximately threefold increase in assembly time.

I suppose that the method of automatic optimization of parameters that takes into account the gene assembly accuracy, implemented in Mabs, can also be applied to other genome, transcriptome and metagenome assemblers.

### Supplementary Information

**Additional file 1**. Supplementary Figures S1 and S2.

## Data Availability

The sequencing reads used in this study are available in the NCBI SRA repository. The SRA accession code for the reads of *Trifolium pratense* is SRR15433789. The SRA accession code for the reads of *Manihot esculenta* is ERR5485301. The SRA accession codes for the reads of *Heracleum sosnowskyi* are SRR23251371, SRR23251372, SRR23251383 and SRR23251384. The SRA accession codes for the reads of *Myripristis murdjan* are ERR3449630, ERR3449634, ERR3449635, ERR3453872, ERR3453873, ERR3453874, ERR3453875, ERR3453876 and ERR3655549. The SRA accession codes for the reads of *Adineta vaga* are SRR13348928 and SRR13348929. The SRA accession codes for the reads of *Mytilus coruscus* are ERR3415816 and ERR3431204. **Availability and requirements** *Project name*: Mabs. *Project home page*: https://github.com/shelkmike/Mabs. *Operating system*: Linux. *Programming language*: Python. *Other requirements*: Python 3, Perl 5, GCC, Zlib-dev, Make. *License*: MIT license. *Any restrictions to use by non-academics*: none.

## Abbreviations

bp: Base pair
Mbp: Megabase pair
Gbp: Gigabase pair
RAM: Random-access memory
PacBio: Pacific Biosciences
CCS: Circular consensus sequencing
AG: The number of accurately assembled genes
C: Complete BUSCO orthogroups
S: Single-copy BUSCO orthogroups
D: Duplicated (or "multicopy") BUSCO orthogroups
F: Fragmented BUSCO orthogroups
M: Missing BUSCO orthogroups
