# Supplementary material for: Mabs, a suite of tools for gene-informed genome assembly
Source: BMC Bioinformatics. 2023 Oct 4;24:377. doi: 10.1186/s12859-023-05499-3 (PMC10548655; doi:10.1186/s12859-023-05499-3)
Supplement: Supplementary file 1 — Additional file 1. Supplementary Figures S1 and S2. [file 12859_2023_5499_MOESM1_ESM.pdf]

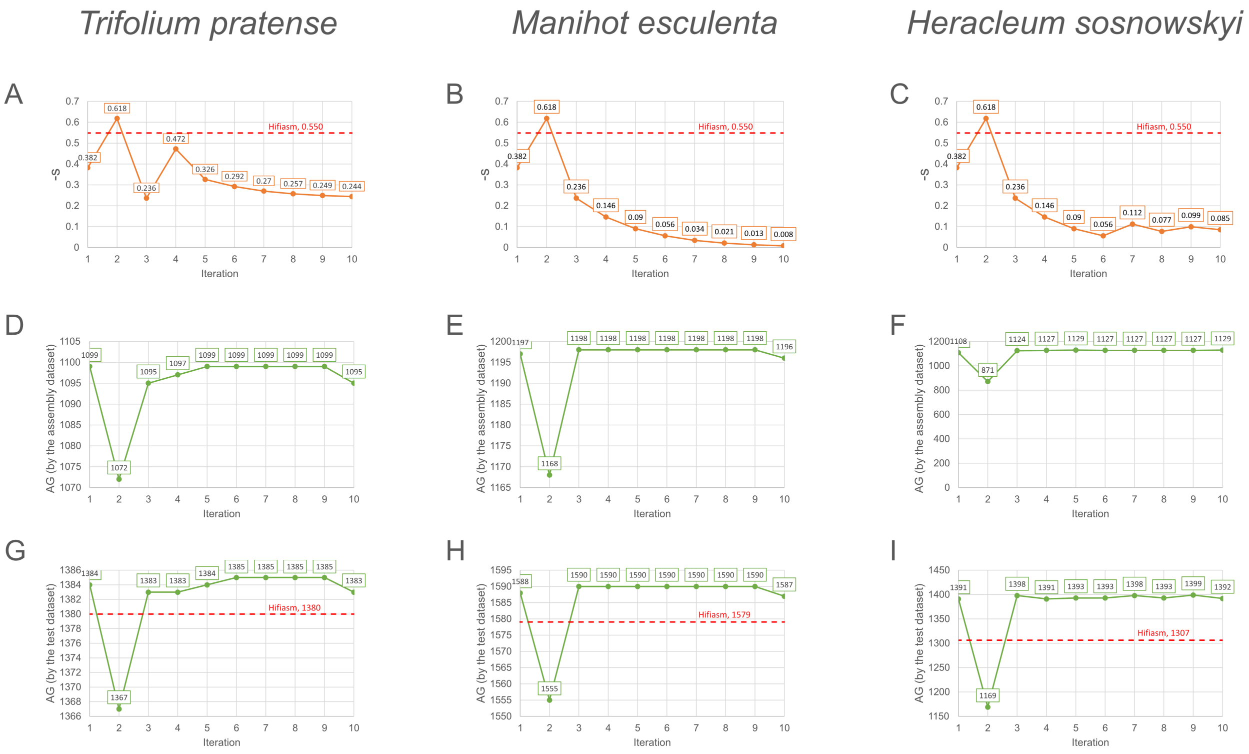

Supplementary Figure S1.
Values of "-s" tried by Mabs-hifiasm during 10 iterations of the golden section search: **A** for *Trifolium pratense*, **B** for *Manihot esculenta*, **C** for *Heracleum sosnowskyi*. The red line denotes the default value of "-s" used by Hifiasm.

AG achieved by Mabs-hifiasm on the assembly dataset (the fourth column in Table 4): **D** for *Trifolium pratense*, **E** for *Manihot esculenta*, **F** for *Heracleum sosnowskyi*.

AG achieved by Mabs-hifiasm on the test dataset (the fifth column in Table 4): **G** for *Trifolium pratense*, **H** for *Manihot esculenta*, **I** for *Heracleum sosnowskyi*. The red line denotes the AG in the assemblies made by Hifiasm without parameter optimization.

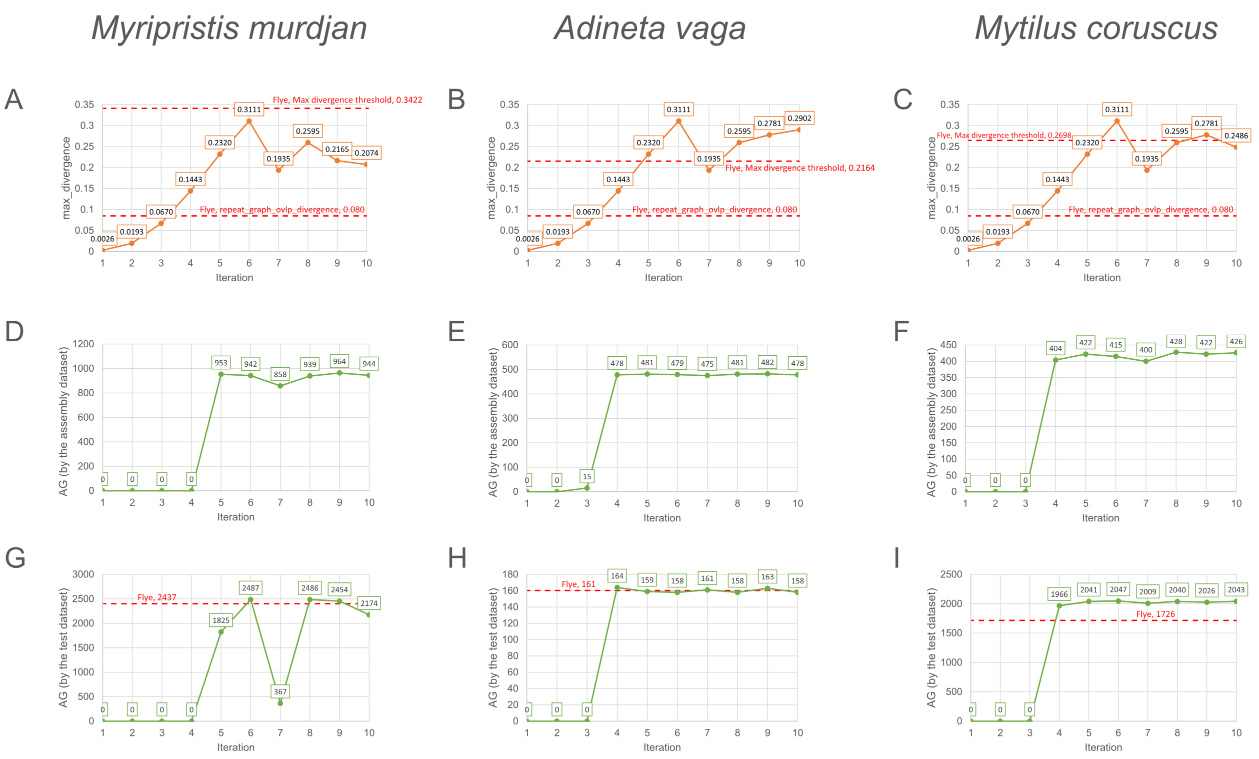

Supplementary Figure S2.

Values of "max_divergence" tried by Mabs-flye during 10 iterations of the golden section search: **A** for *Myripristis murdjan*, **B** for *Adineta vaga*, **C** for *Mytilus coruscus*. Two red lines denote values of "repeat_graph_ovlp_divergence" and "Max divergence threshold" used by Flye; in Mabs-flye these two parameters are equal to each other and called "max_divergence".

AG achieved by Mabs-flye on the assembly dataset (the fourth column in Table 4): **D** for *Myripristis murdjan*, **E** for *Adineta vaga*, **F** for *Mytilus coruscus*.

AG achieved by Mabs-flye on the test dataset (the fifth column in Table 4): **G** for *Myripristis murdjan*, **H** for *Adineta vaga*, **I** for *Mytilus coruscus*. The red line denotes the AG in the assemblies made by Flye without parameter optimization.
